# Evaluation of Different Stabilizers and Inactivating Compounds for the Enhancement of Vero Cell Rabies Vaccine Stability and Immunogenicity:* In Vitro* Study

**DOI:** 10.1155/2019/4518163

**Published:** 2019-03-17

**Authors:** Ebtesam Olayan, Manal El-Khadragy, Aly Fahmy Mohamed, Amany Khalil Mohamed, Rania Ibrahim Shebl, Hany M. Yehia

**Affiliations:** ^1^Chair Vaccines Research of Infectious Diseases, Faculty of Science, Zoology Department, King Saud University, Riyadh, Saudi Arabia; ^2^Zoology Department, College of Science, King Saud University, Riyadh, Saudi Arabia; ^3^Department of Zoology and Entomology, Faculty of Science, Helwan University, Cairo, Egypt; ^4^Holding Company for Production of Sera, Vaccines and Drugs (VACSEA), Egypt; ^5^Department of Microbiology and Immunology, Faculty of Pharmacy, Ahram Canadian University (ACU), Cairo, Egypt; ^6^Department of Food Science and Nutrition, College of Food and Agricultural Sciences, King Saud University, Saudi Arabia; ^7^Department of Food Science and Nutrition, Faculty of Home Economics, Helwan University, Egypt

## Abstract

Inactivation of rabies virus is essential for rabies vaccine preparation where the inactivating compound that is currently recommended for rabies vaccine preparation is *β*-propiolactone (*β*-PL). This compound is considered better than phenol and formalin but it is expensive and potentially carcinogenic. Data revealed that Ascorbic acid (AA) with cupric ions could yield complete and irreversible inactivation of rabies virus without adversely affecting its antigenicity. Additionally, the results of testing the vaccine potency with the selected inactivating compounds were comparable (P<0.05), and ED_50_ was higher than the recommended World Health Organization (WHO) limits. The use of HemaGel (plasma substitute) for testing vaccine stabilization was compared with the currently used vaccine stabilizers (human albumin and lactose). HemaGel yielded better stability than the other tested stabilizers. Monitoring of cellular and humoral immune responses indicated that both the total IgG level against rabies vaccine and the IFN and IL5 levels obtained with the HemaGel-stabilized vaccines were higher than those obtained with human albumin- and lactose-stabilized vaccine candidates.

## 1. Introduction

Rabies is one of the oldest infectious diseases known to mankind. The annual number of human deaths worldwide caused by rabies is approximately 55,000 [1]. An estimated number of 10 million people receive postexposure treatments each year after being exposed to animals suspected to be infected with rabies [2]. Rabies infection results in a rapid progressive encephalomyelitis [3]. The development of the first rabies vaccine by Pasteur successfully reduced the incidence of rabies, but the disease has not been eliminated because it is maintained in many animal reservoirs [4]. Many researchers have attempted to produce an affordable and safe rabies vaccine, and the currently recommended inactivating agent for these neurotropic viruses is beta-propiolactone (*β*-PL), which is very expensive and potentially carcinogenic [5, 6]. Despite *β*-PL keeping the viral antigenicity in the accepted limits other chemicals, such as formaldehyde and phenol, not only inactivate the virus but also adversely influence the viral antigenicity. Therefore, there is a need to identify alternative inactivating agents that are cost-effective and easily available to overcome the known drawbacks of the current inactivants. Ascorbic acid, which is inexpensive and readily available, has also been identified as a possible candidate [6].

Some studies showed that ascorbic acid could inactivate poliomyelitis virus [7], HIV [8], and certain bacteriophages [9]. Another study demonstrated the efficacy of ascorbic acid to reduce the infectivity of herpes simplex virus types 1 and 2, cytomegalovirus, and parainfluenza virus type 2 [10]. Other studies suggested that vitamin C may enhance immune functions, including phagocytosis, neutrophil chemotaxis, and lymphocyte proliferation [11].

Stabilizers are additives that maintain vaccine efficacy despite exposure to heat, light, and other adverse conditions [12]. A locally prepared rabies vaccine has been found to be satisfactorily stabilized with 5% lactose or 2% human albumin [13]. HemaGel was used to stabilize some vaccines and was accompanied by elevated IgG levels [13, 14]. Gelatin is a long-chain molecule that acts as a microsphere for different antigens [15]. It minimizes the macrophage phagocytosis activity that accompanies the decreased release of antigen from the microsphere [16]. As a result, gelatin may be considered an immune modulator that can be used to enhance immune reactivity and stabilize the vaccine [17]. Based on previous findings, the current study aimed to evaluate the viral inactivating potentials of ascorbic acid and *β*-PL as an essential step in the preparation of rabies vaccine, in addition to investigating the impact of using HemaGel as an immune modulator and stabilizer compared to other stabilizers under thermal preservation conditions.

## 2. Materials and Methods

### 2.1. Rabies Virus

Rabies virus strain, the fixed rabies virus Pasteur (FRV), was kindly supplied by Prof Dr. Rifky El-Karamany (the former general manager of research and development sector in the holding company for biological products and vaccines (VACSERA)). FRV was adapted to Vero cells by alternating 37 passages in mice and Vero cells (20 cycles in mice and 17 passages in Vero cells) [13]. The adapted viral strain (designated as FRV/K) exhibited a viral titer of 6.5 log_10_ mouse intracerebral lethal dose 50% (MICLD_50_)/ml and was used to prepare test vaccine.

### 2.2. Vaccine Preparation

Rabies vaccine was prepared in Vero cells using a fixed rabies virus strain (FRV/K) which was adapted to Vero cells. Briefly, Vero cell growth medium was discarded and 5 ml of rabies virus (with a multiplicity of infection of 1-5) was added to each flask. Infected cells were incubated for 1-1.5 h with rotation every 15 min to allow viral distribution and adsorption. Maintenance medium was dispensed into the infected flasks (80-100 ml/flask), and flasks were examined daily using an inverted microscope (Nikon, Japan) to detect cellular changes. Virus-infected medium was harvested at 4 to 5 days of intervals and replaced with fresh medium. Pooled virus harvests were titrated using mice intracerebral inoculation technique, where 10-fold serial dilutions (10^−1^ to 10^−7^) of the viral suspension were prepared in 2% chilled inactivated normal horse serum in phosphate buffer saline (PBS). Each dilution is intracerebrally injected to a group of eight mice (0.03 ml/mice). Mice mortality was recorded for 14 days after inoculation (the count was recorded on day 5 post-inoculation), and the virus infectivity titer was determined according to the procedure outlined by Reed and Muench method [18, 19].

### 2.3. Virus Concentration

Viral suspension was concentrated 10-25-fold using a hollow fiber cartridge and ultra-filtration through membranes with a relative molecular mass cut-off of 100,000. The resulting virus was then pumped through a peristaltic pump.

### 2.4. Virus Inactivation

#### 2.4.1. Beta-Propiolactone (*β*-PL)

The concentrated rabies virus suspension was inactivated using *β*-PL to a concentration of 1/4000 (0.0035 M) according to a previous study [15] and inactivation was performed at 37°C.

#### 2.4.2. Ascorbic Acid (AA)

Ascorbic acid and copper sulphate (Sigma Aldrich, USA) stock solutions containing 0.1 M copper sulphate and 0.5 M ascorbic acid were added to the virus suspension to obtain a final concentration of copper sulphate of 5 *μ*g/ml. Ascorbate concentration was 1 mg/ml. The treated virus was maintained in an incubator at 37°C (Jouan, France). One-milliliter aliquots were collected 2, 4, 6, and 24 h after 48 h of chemical treatment [7].

### 2.5. Evaluation of the Prepared Vaccine

#### 2.5.1. Safety Test

*(a) Toxicity Test. *Eight adult mice and five guinea pigs were inoculated intraperitoneally (I/P) with 0.5 and 1 ml/ animal of the prepared vaccine, respectively. All of the inoculated laboratory animals needed to be free of any signs of toxicity during a 14-day observation period and had to show weight gain.

*(b) Detection of Residual Live Virus. *The prepared vaccine was inoculated intracerebrally (I/C) into five suckling mice families and 20 adult mice. Animals were observed for any signs of rabies for 21 days. To be approved, rabies vaccine must not contain any residual living virus after viral inactivation. Deaths within the first 96 hours were considered insignificant, but the mortality was considered starting from the 5^th^ day post inoculation. The vaccine was classified as safe if all inoculated mice remained alive for a period of 28 days after I/C inoculation [20].

### 2.6. Determination of the Potency of Inactivated Rabies Vaccine

The potency of the inactivated rabies vaccine prepared using different chemical inactivants; (AA and *β*-PL) were evaluated using Mice Immunization Assay (MIA). The effective vaccine dose that protects 50% of infected mice (ED_50_) was measured as follows: both *β*-PL and AA-inactivated vaccines were diluted five-fold (1/5, 1/25, 1/125, and 1/625) in PBS at pH 7.6. Each dilution (0.3 ml) was inoculated intraperitoneally into 16 adult white Swiss male mice, aged 4-6 weeks and weighing approximately 13-16 g. Another set of 16 mice served as negative controls. One week later, the mice were inoculated with a second dose of the vaccine at the same dilution. All mice were challenged intracerebrally 14 days after the first dose of vaccine using the Challenge Virus Standard (Pasteur strain of rabies virus adapted to mouse brain from the Center of Disease Control and Prevention). Challenge Virus Standard (CVS) was prepared to contain 50 lethal dose_50_ (LD_50_; the dose that kills 50 % of mice) of virus per 0.03 ml. Mice were observed for 14 days after challenge, and the number of dead mice or showing signs of rabies (paralysis and convulsions) was recorded in each dilution. Deaths during the first four days after challenge were excluded, and the potency of inactivated rabies virus vaccines was determined according to the WHO protocol for human rabies vaccines by calculating the percentage mortality in each group of mice [18]. ED_50_ was calculated according to Reed and Muench method. Potency of the tested rabies vaccines was expressed in international units (IU) [21].

### 2.7. Stabilizers

Lactose (5%), HemaGel (5%), and human albumin (2%) were used as vaccine stabilizers. Human serum albumin and lactose were sterilized through membrane 0.45 *μ*m filters (Millipore-USA), whereas HemaGel was prepared by steam degradation sterilization of 0.4% gelatin [12–14]. Prepared vaccines were aliquoted as 1 ml/vial.

### 2.8. Immunization of Laboratory Animals

Lactose-, human albumin-, and HemaGel-stabilized vaccines inactivated with *β*-PL and AA were used to immunize six groups of weaning mice (20 mice/ group) through intraperitoneal administration of five doses at days 0, 3, 7, 14, and 28. Another group of mice inoculated with PBS as negative control. Mice were challenged intracerebrally following immunization. Blood samples were collected from mice at 7-, 14-, and 28-day intervals after vaccination and 7-and 14-day intervals after challenge. The use of mice in evaluating rabies vaccine potency and immune response was approved by VACSERA ethical committee. Animal immunization and blood sampling were conducted in class 2 laminar air flow. Scarified and dead animals were decanted according to the safety and occupational health protocol.

### 2.9. Immune Sera Preparation

Collected blood samples were maintained at 37°C for 30 min for blood coagulation then overnight at 4°C for retraction of blood clots followed by cold centrifugation for 15 min at 3500 rpm (Jouan, France). Sera were aliquoted and stored at -80°C until use [8].

### 2.10. Detection of Antibodies against Rabies Virus (RV) Antigen

ELISA plates (Nunc, Denmark) were coated with 100 *μ*l/well rabies antigen in carbonate-bicarbonate buffer (pH 9.6). Plates were incubated for 18 h at 4°C. Unbound antigen was washed out using buffer (PBS-Tween 20). The collected blood samples as well as a reference serum with known concentration were two-fold serially diluted in reciprocal wells. Anti-mouse conjugate labelled with peroxidase enzyme (Sigma Immunochemicals) was added in a final dilution of 1/1000. Plates were incubated for 1 h then unbound conjugate was washed. Tetra methyl benzidine substrate buffer (100 *μ*l) (Sigma-Aldrich, USA) was added. Plates were maintained in dark for 20 min. The reaction was stopped using 100 *μ*l 2 N HCl (Sigma-Aldrich, USA). The developed color was measured at 450 nm using an ELISA reader (Tecan Sunrise, Austria) [22]. Antibody titer was calculated using the following:

(1) Antibody  titer=OD  TestOD  reference×concentration  of  the  reference

### 2.11. Cytokine Determination

High-protein-binding ELISA plates were coated with anti-mouse Interferon gamma (IFN-*γ*) and anti-mouse Interleukin-5 (IL-5) (Biosciences Pharmingen, USA) monoclonal antibodies (100 *μ*l /well; 2.5 *μ*g/ml) against IFN-*γ* and IL-5 cytokines, respectively, for at least 18 h at 4°C in a carbonate/bicarbonate coating buffer (1 L of H_2_O containing 0.07 M Na_2_CO_3_ and 0.173 M NaHCO_3_, pH 9.6). Excess antibodies were washed twice using washing buffer (PBS with 0.05% Tween-20) for 5 min. Antibody-free spots were blocked with 200 *μ*l/well blocking buffer (PBS+0.03% Tween 20+5% non-fat milk) (Kroger, Cincinnati, OH, USA) and incubated for 1 h at 37°C. Plates were washed three times as before. Samples, controls, and standards were dispersed in triplicate (100 *μ*l/well). Tenfold serial dilutions of recombinant cytokines (starting from 2000 pg/ml) were performed to generate a standard curve using assay diluent (PBS+0.03% Tween 20+1% non-fat milk). The assay diluent served as blank. Plates were incubated for 2 h at room temperature then washed three times as previous. Plates were inoculated with 100 *μ*l/well of biotinylated anti-mouse IFN-*γ* and biotinylated anti-mouse IL-5, incubated for 1 h at 37°C, washed, and inoculated with 100 *μ*l/well of 1/1000-diluted peroxidase-conjugated streptavidin (Jackson Immuno Research). Plates were incubated for 1 h at 37°C, washed four times, and inoculated with 100 *μ*l/well of substrate (TMB, Kirkegaard). The reaction was stopped using 100 *μ*l 0.1 N HCl after 10 min of incubation at room temperature. The developed yellow color was measured using UV-Max ELISA plate reader (Molecular Devices Corporation, USA) at 450 nm.

### 2.12. Thermal Treatment

Experimental rabies vaccines stabilized with different stabilizers were maintained at different temperatures (25°C, 37°C, and 40°C) for three months. Potency of each sample was estimated monthly using ELISA.

### 2.13. Statistical Analysis

Each test was carried out in three independent experiments and results were presented as mean ± standard deviation. Data which obtained among different groups were analyzed using one-way Analysis of Variance (ANOVA) and Student's t test.

## 3. Results

### 3.1. Inactivation of Rabies Virus

Recorded data revealed that rabies virus was completely inactivated within 2 hrs after *β*-PL treatment used as 1/4000 final concentrated at 37°C recording a mean depletion of virus infectivity titer in the order of 1.8 log_10_/h mouse intracerebral lethal dose 50% (MICLD_50_) Also, ascorbic acid showed a complete inactivation of rabies virus within 24 h after treatment recording a mean depletion of virus infectivity titer in the order of 1.16 log_10_/h MICLD_50_.

### 3.2. Evaluation of the Prepared Vaccine

Regarding the safety test, all the inoculated animals did not express any signs of rabies toxicity throughout the observation period. In addition, the absence of mortality or signs of toxicity in mice after intracerebral inoculation with test vaccines indicates that the tested vaccines were free from residual living virus.

### 3.3. Determination of the Inactivated Rabies Virus Vaccine Potency (Effective Dose End Point–ED_50_)

Potency test of the inactivated rabies vaccine revealed that the mean effective dose 50% (ED_50_) for *β*-PL- and AA-treated virus was 6.9 ± 0.48 and 6.7 ± 0.65 IU/ml, respectively. In the meantime, there was no significant difference between the inactivation potentials of *β*-PL-inactivated vaccine and AA-inactivated one.

### 3.4. Evaluation of Antibody Level Postvaccination

**Figure 1:**
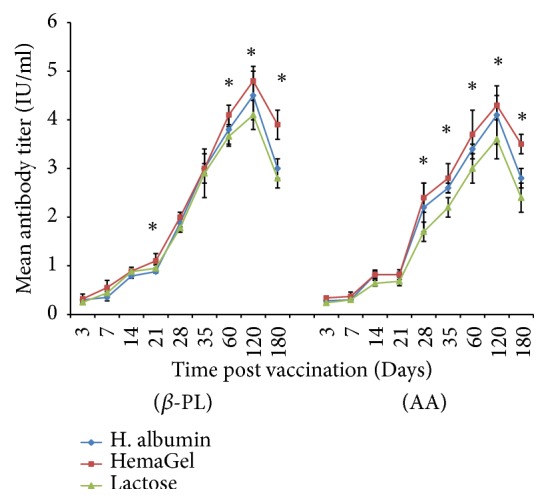
Evaluation of antibody level post immunization of mice with Beta-propiolactone (*β*-PL) and Ascorbic acid (AA) inactivated rabies vaccines stabilized with different stabilizers relatively to time using ELISA. *∗*: statistically significant difference.

Antibody level was compared to a locally prepared reference sample of 2 IU/ml. Data recorded revealed that antibody could be detected 3 days after immunization. Antibody levels were found to be progressively elevated over time. The peak antibody levels were detected 120 days after vaccination (DPV) after immunization using *β*-PL-inactivated vaccine (4.8±0.3, 4.5±0.5, and 4.1±0.3) and AA-inactivated vaccine (4.3±0.3, 4.1±0.6, and 3.6±0.4) in case of HemaGel, human albumin, and lactose stabilized vaccines, respectively. Antibody levels detected after immunization with HemaGel-stabilized vaccine were higher than those detected after immunization with human albumin- and lactose-stabilized vaccines, whereas the lowest antibody level was detected with lactose-stabilized vaccine (P<0.05). Additionally, data revealed that elevated antibody levels obtained after immunization with *β*-PL-inactivated vaccine were slightly higher than those detected postimmunization with AA-inactivated vaccine (P<0.05). Both vaccines were immunogenic (Figure 1).

### 3.5. Cytokine Production

#### 3.5.1. IFN-*γ* Profile

**Figure 2:**
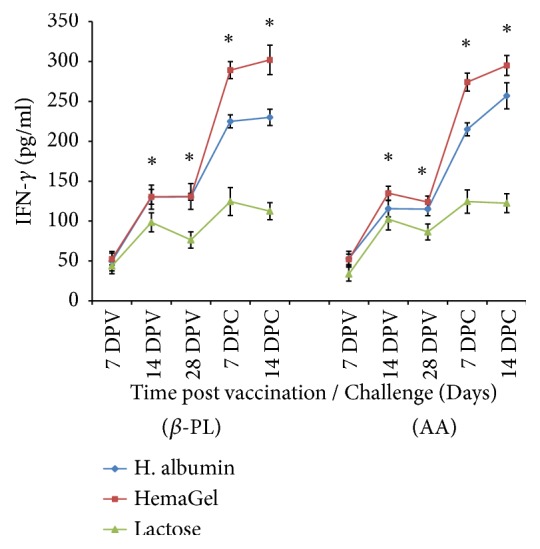
Evaluation of Interferon-Gamma (IFN-*γ*) in mice sera postvaccination with Beta-propiolactone (*β*-PL) and Ascorbic acid (AA) relative to time after vaccination as well as 7 and 14 days after challenge using ELISA. DPV: days after vaccination; DPC: days after challenge. **∗**: statistically significant difference.

*β*-PL- and AA-inactivated rabies vaccines showed detectable IFN-*γ* level 7 days after vaccination. Data clearly showed that HemaGel-stabilized vaccine could significantly induce elevated IFN-*γ* level compared with lactose-stabilized vaccine and an insignificantly increased level compared with human albumin-stabilized vaccine (P<0.05). IFN-*γ* could be traced until the 28^th^ day after immunization with *β*-PL-inactivated vaccines in the order of 130.8±16.2, 130.4±4.3, and 76.4±10.2 and in case of AA-inactivated vaccines by a value of 123.8±7.5, 115±8.2, and 86.4±10 for HemaGel, human albumin, and lactose stabilized vaccines, respectively. A noticeably elevated IFN-*γ* level was detected with maximum value in the 14^th^ day after challenge in case of *β*-PL-inactivated vaccines (302±18.5, 230± 10.2, and 112.4±10.7) and AA-inactivated vaccines (295±12.5, 257±16.3, and 122.4±11.9) that was stabilized with HemaGel, human albumin, and lactose, respectively. It was also obvious that HemaGel-stabilized vaccine induced significantly elevated IFN-*γ* levels compared with other vaccines either stabilized with human albumin or lactose (P<0.001) (Figure 2).

#### 3.5.2. IL-5 Profile

**Figure 3:**
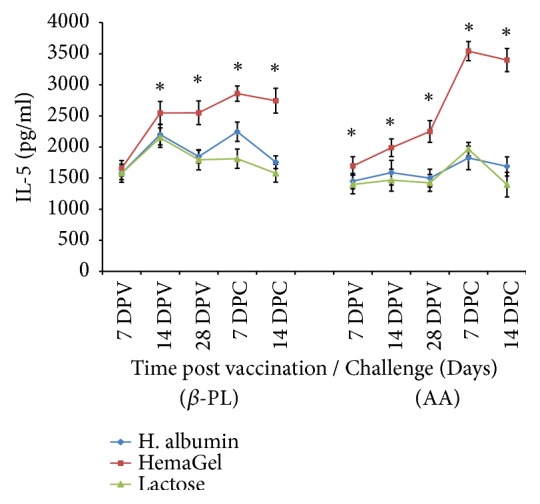
Evaluation of the level of interleukin-5 (IL-5) in mice sera post vaccination with Beta-propiolactone (*β*-PL) and Ascorbic acid (AA) inactivated rabies vaccines relative to time using ELISA. DPV: days after vaccination; DPC: days after challenge. *∗*: statistically significant difference.

HemaGel-stabilized vaccines that were inactivated with either *β*-PL or AA recorded a significant and progressive elevation in IL-5 level relative to time (P<0.05). IL-5 level detected postimmunization using HemaGel-stabilized vaccine was significantly higher than those obtained in case of human albumin- and lactose-stabilized vaccines. The detected IL-5 level in case of HemaGel-stabilized *β*PL-inactivated vaccine (2550±190) was greater than in case of AA-inactivated vaccine (2250±176) until the 28^th^ day after immunization, whereas 7 days after challenge, the elicited IL-5 level reached its peak value and it was greater in case of AA-inactivated vaccine (3542±155) compared to *β*PL-inactivated one (2859±122). On the 14^th^ day after challenge, a significant decrease in IL-5 level was noted in both *β*-PL- and AA-inactivated vaccines stabilized using different stabilizers (P<0.05) (Figure 3).

### 3.6. Stability of *β*-PL and AA Inactivated Rabies Vaccines

**Figure 4:**
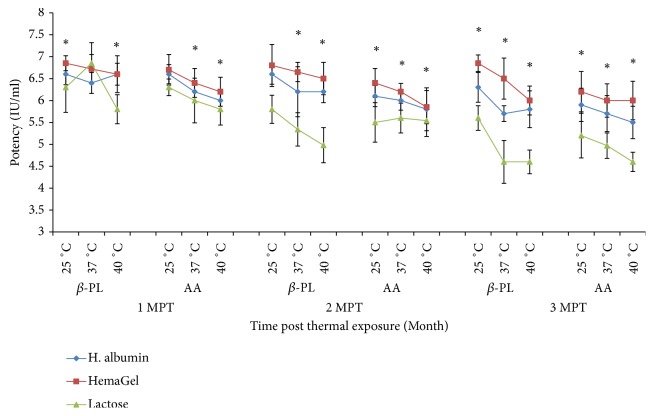
Evaluation of potency of Beta propiolactone (*β*-PL) and Ascorbic acid (AA) inactivated rabies vaccines stabilized with different stabilizers using mice inoculation assay. Vaccine potency was determined one, two, and three months after thermal exposure at 25°C, 37°C, and 40°C. MPT: month after thermal exposure. *∗*: statistically significant difference.

The first, second,and third months after thermal treatment at 25°C data revealed that ED_50_ of lactose stabilized vaccine was significantly depleted compared with ED_50_ of hemaGel and human albumin stabilized vaccines (P<0.05). Stability testing at 37°C showed a convenient stability of hemaGel stabilized one, where ED_50_ of hemagel-stabilized vaccine was insignificantly changed than that of human albumin stabilized one (P>0.05); it was significantly elevated than that of lactose stabilized vaccine (P<0.05). Also, stability evaluation post thermal treatment at 40°C was traced for 3 months and followed similar pattern. HemaGel and human albumin stabilized vaccines were significantly stable and potent than lactose stabilized vaccine over three-month period as indicated by ED_50_ values (P<0.05). Data also revealed that ED_50_ values of the tested vaccines were greater than 2.5 IU/ml (IU/dose) as recommended by WHO (Figure 4).

## 4. Discussion

Rabies virus is a single-stranded negative sense RNA virus that causes fatal encephalitis in both humans and animals [23]. The recommended inactivating agent for this virus is *β*-PL, which is expensive and carcinogenic [6]. It was reported that ascorbic acid with cupric ions is capable of completely and irreversibly inactivating rabies virus as well as other viruses without adversely affecting their antigenicity [6, 23, 24]. Other studies showed that this agent may also inactivate HIV [9] and bacteriophages [10]. For this reason, researchers were interested in using this chemical as antiviral agent.

Although the mechanism of AA viral inactivation was previously described, it remains incompletely understood. Virus inactivation occurs though an oxidative reaction of ascorbic acid catalyzed by cupric ions resulting in the formation of OH groups that inactivate cell-free viruses [10]. In the meantime, rapid inactivating activity of *β*-PL was in agreement with many studies including both DNA- and RNA-viruses [24, 25]. It is important to point out that the current study indicates that ascorbic acid is a good inactivating candidate for rabies virus compared to *β*-PL as both of them did not show residual living virus and they exhibited potential immune response.

ED_50_ values were greater than 2.5 IU/dose and were also in accordance with WHO recommendations as well as another study, which showed an accepted ED_50_ value of rabies vaccine, stabilized with 5% lactose using Vero cells [12].

Vaccine stability study is based on using different stabilizers that can enhance vaccine's thermal stability. Accordingly, current study aimed to evaluate the use of HemaGel to enhance the long-acting stability of locally prepared *β*-PL- and AA-inactivated rabies vaccines as well as its stabilization efficacy under thermal preservation conditions. HemaGel was used as plasma expander to avoid adverse effects of intact gelatin [17] and was utilized as stabilizer [12] to Rift Valley fever Vaccine (RVFV) [26]. This study showed that HemaGel can induce a higher level of RVFV stability and higher long-lasting antibody levels when added to Binary Ethyleneimine- (BEI-) inactivated RVFV than *β*-PL-inactivated vaccine. This finding indicates that the stabilizer and mode of application of the inactivant are important in improving vaccine immunogenicity and potency [7].

HemaGel is an immune-potentiating agent which minimizes the transformational cycle of* Schistosoma mansoni* to the adult stage due to its capability of elevating the antioxidant levels in the infected host cell [27]. The successful use of a live attenuated vaccine depends not only on the proper choice and delivery of the microorganism but also on the maintenance of a sufficient potency to achieve an immune response [28]. Polio virus could be stabilized with gelatin without affecting its virulence [29]. Another study found that the use of gelatin and peptone as stabilizers could maintain the stability of yellow fever vaccine for 3 h at 37°C [30]. Moreover, the shelf life of yellow fever vaccine could be prolonged by thermal preservation at 37°C and 45°C using an accelerated stability method with gelatin and lactose while retaining the vaccine's antigenicity [31]. Newcastle virus strain 12 a vaccine could be also stabilized during storage at 22°C for 38 weeks using 1% gelatin [32].

In the current study, greatest immune reactivity was obtained with *β*-PL- and ascorbic acid-inactivated vaccines stabilized with HemaGel followed by human albumin- and lactose-stabilized vaccines. *β*-PL induced the highest antibody level due to its ability to maintain a vaccine with a better antigenic pattern and limited effect on viral epitope, according to Blackburn et al. [32]. In addition, the concentration required for inactivation depends on the virus type [33, 34]. Consequently, these findings prove the efficiency of hemagel as a potential stabilizer for rabies vaccine under different thermal conditions.

Contrary to our data, a study reported that AA and formalin nonadjuvanted vaccines showed a higher antibody level compared with that detected post immunization with *β*-PL-inactivated vaccine where the oxidation of viral proteins is the inactivating mechanism of AA [26].

In the current study, AA-RV and *β*-PL-RV vaccines showed detectable amount of IFN-*γ* on the 7^th^ day with its peak on the 28^th^ day. AA-RV vaccinated group showed another controversy in IFN-*γ* levels on day 14 after challenge. It was reported that the decrease in the occurrence of viral infections may be obtained by eliciting neutralizing antibody [35–38] where viral clearance is not achieved in absence of IFN-*γ* receptors [39]. Recombinant viral vaccines stimulate Th1-derived immune responses because cytokines such as IFN-*α*, IFN-*γ*, and IL-12 are induced after infection [39]. Other studies reported that Th1 cytokines (IFN-*γ* and IL-2) are detected in serum during infection and after subcutaneous and intramuscular immunization with diphtheria toxin, respectively [40, 41]. In Norway rats, the concentrations of IFN-*γ* and IL-4 were increased and peaked on days 10 to 20 after inoculation with Seoul virus suggesting that both Th1 and Th2 responses are facilitated following infection [42, 43].

IL-5 is mainly produced by activated Th2 and mast cells and acts on B cells to induce proliferation and differentiation into Immunoglobulin- (Ig-) producing cells. IL-5 is an important cytokine, which distinguishes it from the systemic immune compartment [43–46]. IL-5 is promoted in generation of cytotoxic T cells from thymocytes and induces the expression of high-affinity IL-2 receptors. In contrast, murine IL-5 also acts on B-cells and induces their proliferation and secretion of IgM and IgA [47–49] as well as controlling functions of eosinophils and basophils [50].

Evaluation of IFN-*γ* and IL-5 levels from antigen stimulated peripheral blood mononuclear cells of vaccinated individuals is an appreciated tool for exploring cell mediated immune responses following vaccination. That was based on fact that the detection of cytokines such as IFN-*γ* is a proof for type 1 cytokine response that produces Th1 cells. IL-5 is an indicator for type 2 cytokine response which is responsible for the production of Th2 cells. IL-5 is also known for their stimulatory potential to B cells for humoral immune response and its role in promoting immunopathology during viral infections [51].

Recently, new trends for the development of Rabies vaccines were evolved depending on the use of cloned Rabies virus glycoprotein into bacterial plasmids which consequently express the protein in range. An example of theses vaccines is Rabies virus glycoprotein expressed on the surface of Vaccinia virus. Despite the elevated Rabies neutralizing antibody titers elicited in these types of vaccines they were unable to face the existing ones mainly due to issues related to cost as well as the acceptance for human use [52].

In the present study, *β*-PL-RV and AA-RV vaccine-immunized groups showed a detectable amount of IL-5 in sera of immunized mice groups. IL-5 secretion was increased on day 14 and then slightly decreased on day 28 in case of immunization with *β*-PL inactivated vaccine. It has been reported that multiple immunizations over a long period of time result in an enhanced production of IL-5 and IL-10, both of which are associated with Th2 responsiveness [53]. In addition, after immunization with the Attenuated Purified Rabies Vaccine (APRV), induced high levels of IFN-*γ* could be detected in the supernatant of splenocytes, whereas the IL-5 levels were below the detection limit [54]. In consistence with the results of another study, it was demonstrated that immunization with replicating attenuated APRV resulted in detectable cytokine levels of IFN-*γ* but not IL-4 or IL-5 seven days after antigenic stimulation, indicating that the frequency of antigen-specific cytokine-producing cells obtained using APRV was rather low [55].

## 5. Conclusion

Rabies vaccine could be stabilized with the currently available stabilizers as well as with HemaGel. HemaGel proved to be a better vaccine stability enhancer than the currently used stabilizers. In the meantime, AA as inactivant proved to be applicable inactivating agent with almost equal immune potentiating activity as that of current recommended *β*-PL.

## Data Availability

The data used to support the findings of this study are included within the article.
